# 249. Evaluation of 99 Radiologically-proven Osteomyelitis Cases

**DOI:** 10.1093/ofid/ofab466.451

**Published:** 2021-12-04

**Authors:** Olcay Buse Kenanoğlu, Gunel Quliyeva, Tansu Yamazhan, Bilgin Arda, Meltem Taşbakan, Hüsnü Pullukçu, Hilal Sipahi, Şöhret Aydemir, Sercan Ulusoy, Oğuz Reşat Sipahi

**Affiliations:** 1 Ege University Faculty of Medicine, İzmir, Izmir, Turkey; 2 Professor Doctor, Izmir, Izmir, Turkey; 3 Dr, izmir, Izmir, Turkey

## Abstract

**Background:**

Herein we aimed to evaluate osteomyelitis cases in our setting.

**Methods:**

We evaluated the hospital records of patients with osteomyelitis between January 2013 and December 2020 retrospectively. Osteomyelitis was confirmed by direct radiography or magnetic resonance imaging or pathology. Demographic features, risk factors, clinical/laboratory findings, treatment response and mortality rates were evaluated. Clinical response was defined as (resolution of clinical signs including fever and purulent discharge and other symptoms) and/or negative culture at the end of antimicrobial therapy.

**Results:**

Patients were 33 female, aged 29–85 years (mean 59±12.6). Fourty nine of the patients were diabetic foot infection, 30 were spondylodiscitis, eight were primary, seven were post-traumatic, and five were post-surgical osteomyelitis. Overall 62 patients had diabetes mellitus and 16 patients had chronic renal failure. Peripheral arterial disease, neuropathy, diabetic retinopathy and venous insufficiency rate in the DM subgroup is shown in table. Fever was present in 24.2% of the cohort. İncreasing of CRP was in 95,9%, erythrocyte sedimentation rate in 83,9%, and leukocytosis in 37.3%. The radiological findings of osteomyelitis were detected via magnetic resonance imaging in 73 patients. Etiology in biopsy cultures were elucidated in 59.5% and the most common pathogen was *S. aureus* (30%) Table1. The most common empirical treatment regimens were tigecycline in 27 patients, ampicillin/sulbactam in 19 patients and ceftriaxone+teicoplanin in 12 cases. Duration of treatment was 36,2±17.3 days (range 6-104 days). Overall, clinical response was obtained in 91.9%. Fifty patients were performed surgical procedure + antibacterial treatment, clinical response was 96% (p:0.091). Surgical debridement could be performed in 22 patients, clinical response was obtained in all (p:0.193). Thirteen patients developed recurrence within one year. Sixty-seven patients received oral consecutive treatment after discharge. In hospital mortality rate was 2/99 (2,02%).

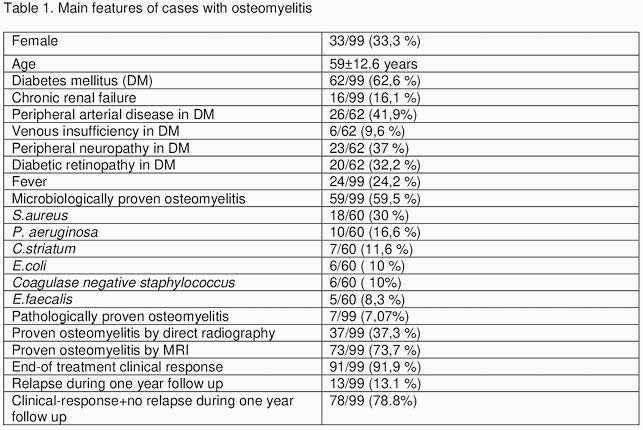

**Conclusion:**

Despite surgical debridement and/or developed antimicrobial treatment, approximately 1/5 of osteomyelitis cases required further treatment Further interventions seem to be needed to reach better outcomes.

**Disclosures:**

**All Authors**: No reported disclosures

